# 31-year contrasting agricultural managements affect the distribution of organic carbon in aggregate-sized fractions of a Mollisol

**DOI:** 10.1038/s41598-020-66038-1

**Published:** 2020-06-03

**Authors:** Ming Sheng, Xiaozeng Han, Yihe Zhang, Jinghong Long, Na Li

**Affiliations:** 1 Key Laboratory of Mollisols Agroecology, Northeast Institute of Geography and Agroecology, Chinese Academy of Sciences, Harbin, 150081 P.R. China; 2 University of the Chinese Academy of Sciences, Beijing, 100039 P.R. China

**Keywords:** Carbon cycle, Agroecology

## Abstract

Evaluation of soil organic carbon (SOC) dynamics is often limited by the complexity of soil matrix. Quantitative information on the distribution of SOC within aggregate hierarchy will help elucidate the carbon flow in soil matrix. However, this knowledge still needs to be documented. Soils were sampled from a surface Mollisol with plots under 100 years of continuous cropping, 31 years of simulated overgrazing to severely degraded bareland, and grassland restoration from cropped soil. A combined density and chemical fractionation procedure within water-stable aggregate was utilized to quantify the distribution of OC after long-term different land use patterns. Results showed that grassland significantly increased total SOC and mean aggregate associated OC compared to initial soil in 1985 with total SOC (g kg^−1^ soil) from 46.1 to 31.7 and mean aggregate associated OC (g kg^−1^ aggregate) from 31.6 to 44.7. Converting cropland to grassland also enhanced the formation of macroaggregates (>0.25 mm) (from 34.7% to 52.2%) and increased the OC concentrations in density and humic fractions by 48.3%-75.9% within aggregates. But the proportions of OC in density and humic fractions to SOC only increased in macroaggregates in grassland. Alternatively, converting cropland to bareland caused substantial depletion of total SOC, macroaggregates and their associated OC concentrations. The SOC (g kg^−1^ soil) and mean aggregate associated OC (g kg^−1^ aggregate) significantly decreased from 31.7 to 25.7 and from 31.6 to 26.2, respectively. While the OC concentration of density and humic fractions within aggregates in bareland did not show significant decreases. Principal component analysis demonstrated that the soils were developed by contrasting land use changes, with the grassland soil being more associated with labile OC fractions within macroaggregats and bareland soil more associated with recalcitrant OC fractions within microaggregates and silt-clay units. These findings highlighted the favorable preservation of plant-derived carbon within soil aggregates, particularly in the labile OC fractions within macroaggregates under high plant inputs with 31 years of grassland conversion. For the cropland and bareland soils without organic inputs, more OC was stabilized within fine aggregates via organo-mineral interactions, tending to be more recalcitrant.

## Introduction

The processes governing the formation, transformation, and stabilization of soil organic carbon (SOC) are closely tied to soil aggregate architecture^1^. Soil aggregates can physically protect SOC against decomposition and store SOC crucial to climate change mitigation^2^. Also, the location of organic carbon within aggregate is a key factor for the stabilization of SOC^3^ due to spatial arrangement of soil constituents and soil microbes^1,4^. One important but poorly understood aspect of soil organic matter (SOM) decomposition and stabilization is the role of soil aggregates. Soil aggregates controlled the degree of hierarchical inaccessibility of SOC to decomposing organisms or catalytic enzymes^5,6^, the availability of SOC and nutrients, and their responses to land-use changes^7,8^. Better understanding of the aggregate-associated carbon, as well as the carbon allocation within aggregates, are crucial for SOC sequestration and stability^9–11^. Whether SOC accumulates or decreases depends largely on the interactions between aggregate architecture and soil biota affecting SOC decomposition and/or stabilization^12^.

SOC decomposition rate reduced within soil aggregates in comparison to bulk soil; the interactions between SOC dynamics and aggregate turnover were affected by organic manuring and other changes in land use managements varying in the quantity and quality of organic matter entering soil^9,13^. A relatively large proportion of organic residue was accumulated in the coarse fraction within macroaggregates, but the OC in microaggregate and silt-clay units did not continue to increase with additional organic manure inputs^14^. These results suggested that the OC saturation occurred in a hierarchical fashion. The silt-clay unit saturated before larger sized soil aggregates with increasing organic inputs. As for different land use patterns, the aggregate-associated OC increased after conversion of forest to natural grassland due to anthropic disturbance reduction, especially in macroaggregates^15^. While other studies reported that natural grassland increased the OC more significantly in microaggregates than that in macroaggregates^12^. Soil tillage after converting grassland to cropland could cause loss of C-rich macroaggregates, particularly free fraction, thus resulting in increases of C-depleted microaggregates^8,16^. The discrepancy may be related to the complex nature of SOM as well as the methods used to characterize SOC fraction changes during residue decay, aggregate turnover, and SOM formation^17^. Physical and chemical fractionation method has been proposed as a useful tool for investigating SOC dynamics due to its linkage of stabilization mechanisms of OC in soil aggregate^18,19^. In addition, the aggregate-associated OC of various stability and mineralogical compositions have diverse structural and functional C groups representing distinct roles in organic matter dynamics^18,20^. For example, the OC in microaggregate and silt-clay units were believed to be more stable and recalcitrant than that in macroaggregate and coarser fractions^21^. Recently deposited OC preferentially accumulated in the free fraction and contributed to the formation of macroaggregates, eventually being redistributed among aggregate classes through the destruction and re-formation of aggregates^8,22^. Furthermore, the SOC concentrations within aggregates were hypothesized to increase with the increasing size of aggregates according to the hierarchy theory^23,24^; the responses of SOC to different land use patterns were related to changes in SOC associated with different aggregate fractions^25,26^, though this is still under debate^5^. Previously, long-term field experiments investigating SOC dynamics under different land use patterns have shown contrasting effects related to the SOC fractionation methods^11,22,27^. Combining the physical and chemical fractionation methods within soil aggregates may assist in determining the effects of long-term land use change on the physicochemical sequestration mechanisms of OC in specific soil matrixes^3,18^.

Mollisols, famous for high SOC and a well-structured compartment, are mainly located in northeastern China with a total area of 7.0 × 10^6^ ha, of which about 69% was cultivated^28^. They were once very fertile before reclamation. The SOC content has declined by 65% due to extensive cultivation and other anthropogenic activities^29^. This soil degradation has endangered sustainable crop production and even national food security. Therefore, suitable soil management increasing SOC and improving soil quality in this region are being fully considered. Perennial ecosystems, such as grassland or conversion of grassland from cropland, have been proven as more efficient than permanent cropping systems in terms of C distribution, C sequestration, and increasing nutrient availability of the agroecosystem in Mollisols^30,31^ and other soils^27,32^. A deeper follow up of OC accrual or fractionation regimes after introduction of perennial grassland from cropping systems needs to be carried out with a combination of physical and chemical fractionation procedure within soil aggregates. We hypothesized that perennial grassland could improve SOC at the mid- to long-term due to continuous organic residues entering into the soil. Furthermore, we hypothesized that changes in land-use patterns could also influence the OC allocation in specific compartments of the soil matrixes, though the overall effect was even hidden when bulk soil was analyzed.

In this study, we reported the results of a combined fractionation scheme where density and humic fractions within water-stable aggregates were separated from surface Mollisols under 31-year contrasting land use patterns. The field experiment consisted of soybean-maize-wheat rotation under 100 years of continuous cropping, 31 years of simulated overgrazing to severely degraded bareland, and grassland restoration from cropped soil. The presence of bareland allowed comparison of persistence of SOC and OC allocation within soil aggregates in the absence of fresh inputs and soil perturbation against the other two different treatments. The aims of this study were to: (1) quantify and compare the OC changes in density and humic fractions within aggregates of different sizes, (2) determine the effects of contrasting land use patterns with different quantity and quality of organic inputs, and (3) try to elucidate the stabilizing mechanisms of OC within soil aggregate matrixes.

## Results

### Soil properties in bulk soil

Over 31-year different land use changes, soil pH was higher in grassland than in bareland and cropland soils, but showed no significant differences compared with soil in 1985. Soil bulk density (SBD) decreased significantly from 1.05 g cm^−3^ to 0.90 g cm^−3^, by 14.3% in grassland, but the SOC and TN contents significantly increased, from 31.7 g kg^−1^ to 46.1 g kg^−1^ and 3.0 g kg^−1^ to 4.0 g kg^−1^, respectively (*P* < 0.05). Cropland and bareland showed the opposite changes, with the changes in bareland soil being larger than those in cropland. The distribution of particle-size fractions did not change significantly over 31-year different land use patterns, with an average value of 26.0% for the 0.02-2 mm fraction, 30.7% for the 0.002-0.02 mm fraction, and 41.7% for the <0.002 mm fraction. The soil microbial biomass carbon (SMBC) followed the order of grassland > cropland > bareland, and the SMBC in grassland were 1.8 and 2.3 times higher than in cropland and bareland soils, respectively (Table 1).

**Table 1:** Selected soil properties at 0–10 cm depth in the initial soil in 1985 and soils after 31-year different land use patterns.

Soils	Soil pH	Soil bulk density (g cm^−3^)	Particle size distribution (%)			Soil organiccarbon (g kg^−1^)	Total nitrogen (g kg^−1^)	Soil microbial biomass C (mg kg^−1^)
			Sand 0.02–2 mm	Silt 0.002–0.02 mm	Clay <0.002 mm			
Soil in 1985	6.20 ± 0.08ab	1.05 ± 0.03a	25.2 ± 2.6a	31.2 ± 0.9a	43.6 ± 2.1a	31.7 ± 0.7b	3.0 ± 0.1b	—
Cropland	6.05 ± 0.06b	1.06 ± 0.02a	25.5 ± 0.6a	30.6 ± 0.8a	41.6 ± 1.8a	29.1 ± 0.5c	2.5 ± 0.1c	283 ± 21b
Bareland	6.08 ± 0.04b	1.13 ± 0.05a	26.4 ± 0.4a	30.4 ± 1.0a	41.2 ± 1.4a	25.7 ± 0.5d	2.1 ± 0.1d	218 ± 4b
Grassland	6.42 ± 0.13a	0.90 ± 0.06b	26.2 ± 1.3a	31.2 ± 1.1a	42.3 ± 1.6a	46.1 ± 0.6a	4.0 ± 0.1a	506 ± 50a

Values are means ± standard deviation (n=3). Different lowercase letters indicate significant differences among soil in 1985 and field treatments at P < 0.05.

### Organic carbon in water-stable aggregates

**Figure 1:**
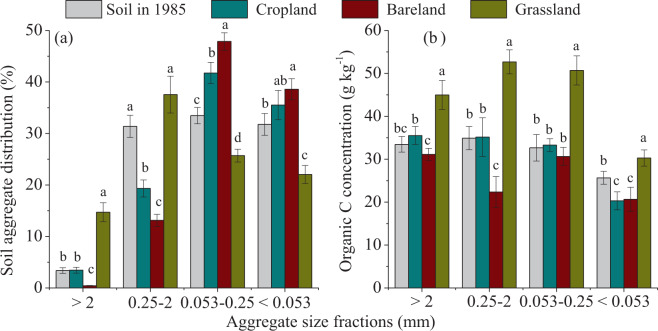
Soil aggregate distribution (**a**) and organic carbon concentrations in aggregates (**b**) in soil in 1985 and different land use treatments. Different lowercase letters in the same aggregate size group indicate significant differences among soil in 1985 and field treatments at *P* < 0.05.

Compared with the initial soil in 1985, the weight percentages of >2 mm large macroaggregate and 0.25-2 mm small macroaggregate dramatically increased from 24.7% to 52.2% in grassland, but the percentages of 0.053-0.25 mm microaggregate and <0.053 m silt-clay fraction decreased. On the contrary, 31-year cropping and bareland caused decreases in >0.25 mm macrroaggregates and increases in <0.25 mm aggregates, with larger changes in bareland than in cropland soil (Fig. 1a). 31-year grassland significantly increased the OC concentration in all aggregate fractions, with the mean aggregate associated OC (g kg^−1^ aggregate) from 31.6 to 44.7, and higher increments were observed in 0.25-2 mm and 0.053-0.25 mm aggregate fractions, the increment was lowest in the silt-clay unit (*P* < 0.05) (Fig. 1b). Converting cropland to bareland caused substantial depletion of macroaggregates and their associated OC concentrations. The percentage of macroaggregate (%) and mean aggregate associated OC (g kg^−1^ aggregate) significantly decreased from 34.8 to 13.6, and from 31.6 to 26.2, respectively. Bareland only caused significant decrease of the OC concentration within 0.25-2 mm macroaggregate compared with soil in 1985 (*P* < 0.05). Cropland caused no significant changes of OC concentrations in all aggregate sizes, being higher in macro- and microaggregates, and lower in the silt-clay unit compared with soil in 1985. The aggregate-associated OC concentrations in all aggregates were significantly higher in grassland, followed by cropland, and lowest in bareland, significantly higher OC was also found in >0.25 mm macoaggregate in cropland than that in bareland (*P* < 0.05) (Fig. 1b).

### Organic carbon of density and humic fractions within aggregates

**Figure 2:**
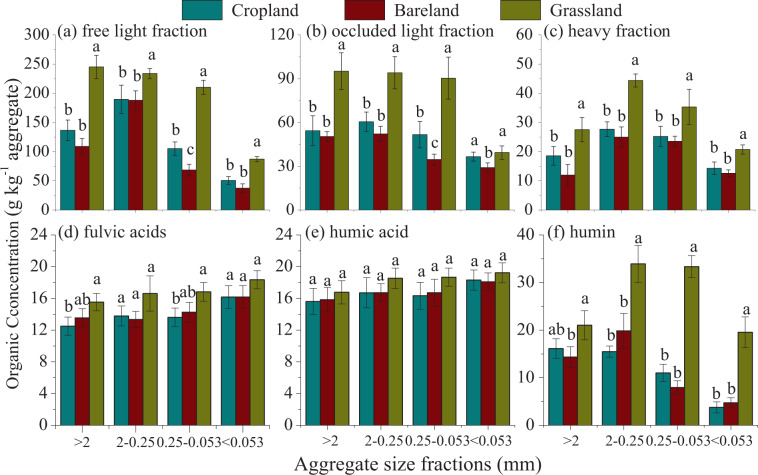
Organic carbon concentrations of density (**a**–**c**) and humic fractions (**d**–**f**) within soil aggregate fractions after 31-year different land use patterns. Different lowercase letters in the same aggregate size group indicate significant differences among treatments at *P* < 0.05.

The OC concentrations of different density and humic fractions differed obviously among soil aggregate hierarchy and land use patterns. Specifically, within all aggregate fractions, the OC concentrations of all density and humic fractions were highest in grassland, followed by cropland and bareland. Also, the OC concentration showed no significant differences in cropland and bareland soils among aggregates, with the exception of higher OC of free light (fLF) and occluded light (oLF) fractions within microaggregate in cropland than those in bareland soil (*P* < 0.05). The free light (fLF) and occluded light (oLF) fractions contained obviously higher OC concentrations than those in heavy fraction (HF) and humic fractions (Fig. 2). Among all the field treatments, the OC concentrations of density fractions were highest within 0.25-2 mm aggregates, and then decreased with the size of the aggregates, with the <0.053 mm silt-clay unit having the lowest OC concentrations (Fig. 2a–c). The OC concentrations of humic fractions within aggregates did not show the same changing treads as density fractions after 31-year land use changes. There were no significant differences of OC concentration of fulvic acids (FA) and humic acids (HA) among field soils, with the exception of higher OC concentrations of FA in >2 mm and 0.25–0.053 mm aggregate fractions in grassland than in cropland. The OC concentration of humin (HU) showed similar changes as HF, significantly higher OC concentrations were found within 0.25–2 mm and 0.053–0.25 mm aggregates in grassland (Fig. 2d–f).

**Figure 3:**
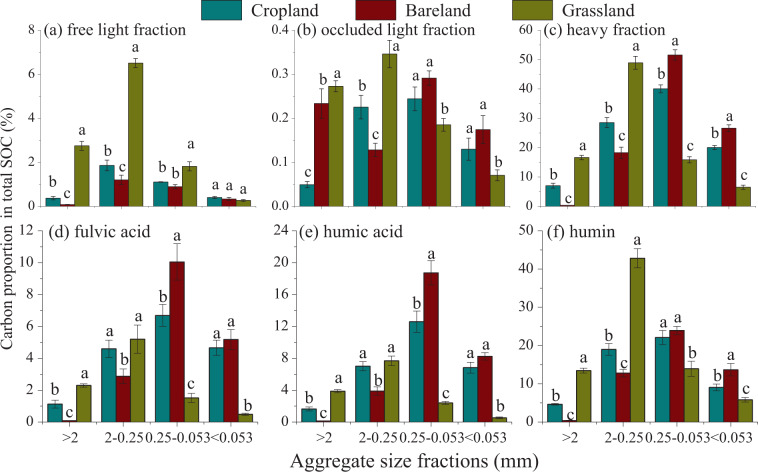
Proportions of organic carbon of density (**a**–**c**) and humic fractions (**d**–**f**) to total SOC within different sized aggregates after 31-year different land use patterns. Different lowercase letters in the same aggregate size group indicate significant differences among treatments at *P* < 0.05.

The proportions of density- and humic-associated OC in SOC differed obviously with OC concentration among land use patterns and soil aggregate hierarchy (Fig. 3). Among all the field treatments, the HF and HU fractions, containing lower OC concentrations, were the most abundant OC fractions relative to total SOC. The highest proportions were found within 0.25-2 mm aggregate in grassland and 0.053-0.25 mm aggregate in cropland and bareland (Fig. 3). Specifically, within >2 mm and 0.25-2 mm macroaggregate fractions, the proportions in SOC were significantly higher in all density and humic fractions in grassland than in the respective fractions of both cropland and bareland soils (*P* < 0.05). The bareland soil had the lowest OC proportions in density and humic fractions, with the exception of higher occluded light fractions (oLF) within >2 mm macroaggregate in bareland than that in cropland (*P* < 0.05). Within 0.053-0.25 mm and <0.053 mm aggregates, higher OC proportions were found in bareland soil, followed by cropland, and grassland had the lowest proportion except for higher OC proportion of fLF in grassland than that of cropland and bareland (Fig. 3).

### Interrelations

**Figure 4:**
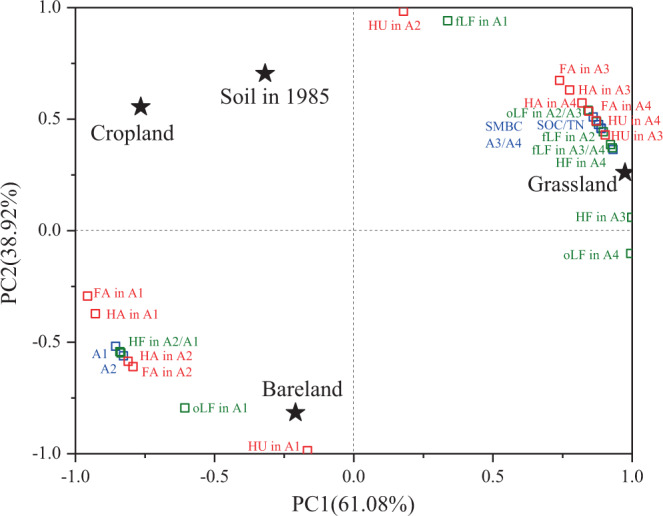
Principle component analysis (PCA) including all organic carbon fraction in bulk soil and within different sized-aggregate fractions in soil in 1985 and field soils with three land use patterns. The black stars represent the soil in 1985 and three field soils; the blue, green, and red hollow squares represent the organic carbon in different fractions in bulk soils, density fraction within aggregates, and humic fractions within aggregates, respectively. SOC, soil organic carbon; TN, total nitrogen; SMBC, soil microbial biomass carbon; fLF, free light fraction; oLF, occluded light fraction; HF, heavy fraction; FA, fulvic acid; HA, humic acid; HU, humin; A1, <0.053 mm aggregate; A2, 0.053–0.25 mm aggregate; A3, 0.25–2 mm aggregate; A4, >2 mm aggregate.

Principal component analysis (PCA) including all aggregates in bulk soil, density, and humid fractions within all aggregate sizes, demonstrated significant effects of field treatment and soil aggregate hierarchy via both PC1 and PC2, which explained 61.08% and 28.92% of the total variance, respectively (Fig. 4). The grassland, cropland, and bareland soils separated from the soil in 1985 in different directions. It developed in two contrasting directions via PC1, the cropland and grassland were divided by the soil in 1985, with cropland more similar to the soil in 1985. The bareland soil was separated from soil in 1985, cropland and grassland soils via PC2. The separation towards grassland via PC1 was associated with SOC, TN, and SMBC, >0.25 mm macroaggregate and related density and humic fractions within macroaggregates, fLF and oLF fractions within microaggregates, and fLF within the silt-clay units. The separation towards cropland via PC1 was mostly driven by the mass distribution of microaggregate and the silt-clay unit, and OC in HF, FA, and HA fractions thereof. The separation towards bareland via PC2 was mostly driven by HU within microaggregates and the silt-clay units, and fLF and oLf within the silt-clay units (Fig. 4).

## Discussion

### Plant residue inputs: the driving factors linking SOC dynamics and land uses

The soil in 1985 had been used for more than a century for cropping, it was utilized to explain the changes of soil properties after 31-year of continuous cropping. Over 31-years continuous cropping, the selected soil properties did not show as much change as in natural grassland and bareland soils, with SOC and TN contents decreased compared with the soil in 1985 (Table 1). Such changes were consistent with previous results showing that long-term continuous cropping generally decreased SOC content^33^. The cropland soil here was considered as the “reference” soil when determining the C redistribution within soil aggregates under contrasting land use patterns. The changes in OC of density and humic fractions within aggregates were interpreted both on the OC concentrations per mass aggregate and on OC proportion of each fraction relative to the total SOC of the whole soil base by comparing the continuous cropping in cropland to the absence of vegetation in the bareland and conversion to natural grassland with high level of plant residue input in grassland.

In natural grassland, the estimated shoot and root biomass were 4-9 t ha^−1^ and 12-43 t ha^−1^, respectively. The highest amount of root biomass (i.e. root density) was also found in the upper 10 cm for the grass species of the present experiment (data not published). However, there were no, or only limited, OC inputs from root exudates in bareland and cropland. Higher amounts of plant residue inputs from natural grassland were theoretically the dominant factor influencing the SOC dynamics and OC redistribution patterns in soil matrixes as shown in our results^27^ (Figs. 1–3). In addition, more significant increments of SOC and TN contents, macroaggregates, and associated OC concentrations were found for bulk surface soil in grassland than in bareland and cropland soils (Table 1; Fig. 1). This indicated that the vegetation type (natural perennial versus crops) and organic C inputs from above- and below-ground plants (only observed in grassland soil) could promote the accumulation of SOC and soil aggregation, as has been reported in Mollisol^31^ and other soils^34^. It was the accumulation of relatively large amounts of plant residue in grassland, with the unique climate conditions in the Mollisol region, that contribute to the relatively high accumulation of SOC in grassland^29^ (Table 1). Nevertheless, the SOC, TN, and SMBC contents decreased in bareland soil, indicating a sharp loss of labile OC in soil likely due to long-term substrate limitation and exhaustion with no OC amendments^35^.

### Soil aggregates response to land use patterns

Soil aggregate size distribution varied significantly in terms of land use types (Fig. 1a). The macroaggregates (>0.25 mm) distribution, which generally had an order of grassland > initial soil in 1985 >cropland >bareland, has been observed to decrease or disappear with sustained planting^36^. Converting cropland to perennial grassland 31 years ago caused increases in large macroaggregate and small macroaggregate of 269.7% and 164.5%, respectively, but decreases of 43.3% and 58.8% in microaggregate and silt-clay unit, respectively. In grassland soil, continuous plant residue inputs facilitated the aggregation of soil particles causing micro-aggregates to form as macro-aggregates^36^, thus increasing the larger sized aggregates and decreasing the smaller sized aggregates^24,37^. Additionally, the mass percentage of large and small macroaggregates dropped dramatically in bareland soil compared to grassland soil (Fig. 1). The disruption of soil aggregates may expose once-protected organic matter to decomposition^38^ and induce organic C loss in soil, as has been observed here (Fig. 1). In addition, wind and water erosion may destroy soil macroaggregates, causing loss of aggregate-protected C since there are no plants growing in the bareland soil^39,40^. Continuous cropping also caused decrease of small macroaggregate, due to annual soil tillage at the surface 0-20 cm soil depth. Soil tillage could destroy soil macroaggregates and cause the redistribution of OC for the arable perturbed plot under maize-soya bean-wheat rotation^38^.

Soil aggregate-associated OC within different aggregate hierarchies can provide critical insight into C sequestration mechanisms and their contribution to SOC dynamics^1,36,41^. In line with previous studies, our results showed that the aggregate-associated C varied across aggregate sizes and land use patterns^9,42–44^. The increases of OC concentrations were observed in all aggregate fractions in grassland (Fig. 1b). Also, larger increments of OC concentrations were detected for fast-responding aggregate fractions, such as the large macroaggregate (>2 mm) and macroaggregate (0.25-2 mm) (Fig. 1b). This demonstrated that the plant-derived C entered into soil to form SOM, and macroaggregate played a more dominant role than microaggregate and silt-clay unit in C sequestration, as has been reported elsewhere^5,8,14^. The improvements were expected because the no-tilled perennial restoration led to aggregation of fine soil particles or the increase of root exudates by adding large amounts of plant residue into the soil^6,33,44^. The no-tilled perennials facilitated the protection of SOM from microbial degradation in different aggregate-size classes due to lower soil disturbance, which in turn favored the generation of physically stable large and small macroaggregates^43^. In addition, the new OC sources derived from recently photosynthesized products such as plant residue and root biomass play evidential roles in driving biological processes in soil^45^. Sufficient nutrient availability in grassland soil enhanced soil microbial processes and further caused SOC accumulation via soil microbial residue^30^. Indeed, there were more macro-aggregates and associated organic C in grassland than cropland and bareland (Fig. 1). Continuous cropping slightly increased the aggregate-associated OC concentrations in macro and microaggregates, but significantly decreased the OC concentration in the silt-clay unit (*P* < 0.05). Soil tillage in cropland induced soil C loss by acceleration of organic C decomposition^46^. The acceleration of organic matter decomposition was at least partly due to the disruption of the soil macroggregates, which exposed once-protected organic matter to decomposition^38^. In bareland, the OC concentrations decreased in all aggregate fractions compared with those in soil of 1985 (*P* < 0.05) (Fig. 1b). Because the bareland soil neither received OC amendments nor had plants grown for up to 31 years, this caused limited nutrient availability for soil microorganisms, reducing the physical protection capacity and causing decomposition of old C of soil aggregates^47^ (Fig. 2).

### Organic carbon allocation within aggregates under different land use patterns

Soil aggregates, serving as “sieves”, protected C by providing physical barriers between OC and OC decomposers, thus influencing the OC distribution and stabilization thereof^1,5,35,41,48^. In this study, the combined density and humic fractionation method of SOC could help quantify the allocation regimes of OC within aggregates and their underlying sequestrating mechanisms within soil matrixes^3,17^. The OC distribution varied within different sized aggregates under land use and agricultural managements due to changes of nutrient availability, soil biota, and their interactions with soil aggregates^48,49^. Compared with cropland and bareland soils, relatively higher OC concentrations were accumulated in all density and humic fractions within all aggregate sizes in grassland. Also, the proportions of OC in density and humic fractions to total SOC were higher in large and small macroagregates in grassland while the proportions of OC in density and humic fractions in microaggregates and silt-clay units were lower in grassland than those in cropland and bareland soil (Fig. 3). This suggested that not only plant residue (only found in grassland soil) were supposed to enhance C accumulation in macroaggregates, but also did so for vegetation type (perennial versus annual crops), reduction in tillage, or a combination. Previous studies reported that soil receiving higher organic inputs increased the coarse fraction within macroaggregate^14,25,48^. The coarse or labile C fraction within macroaggregates may not only be lost temporally but also could be restored rapidly upon conversion of cropland into grassland, and the new steady status of OC can occur earlier in the coarse fraction than in bulk soil due partly to microbial residue^30,50^. In particular, prominent increases of OC proportions in the density and three humid fractions were observed in 0.25-2 mm macroaggregates in grassland soil compared to cropland and bareland soils (Figs. 2, 3). This 0.25-2 mm macroaggregates in grassland were considered to be the “preferential aggregate fractions” where most of the plant residue C was accumulated (Fig. 1b, 2 and 3) and can be used as an indicator of management effects^15,37^. Our results were in line with others^21,22^, identifying a gradient of OC incorporation into water-stable aggregates with faster response to land-use change by macroaggregate fractions compared with microaggregates and silt-clay units. The PCA analysis also confirmed these findings, in which the OC fractions within macroaggregates in grassland were shifted on the right plot from cropland and bareland soils (Fig. 4).

The proportions of OC in total SOC decreased in density and humic fractions within macroaggregates, and increased within microaggregates and silt-clay units in bareland soil without any fresh OC amendments and in cropland soil with only limited root exudates (Fig. 3). This indicated a predominant loss of labile and decomposable organic matter in the macroaggregate fraction likely due to long-term substrate limitation and soil erosion^51^. Also, the 0.25-0.053 mm microaggregates was considered as the “preferential aggregate” in cropland and bareland soils. The cropland and bareland soils lacked new plant-derived C for up to 31 years resulting in deficient C and nutrient sources for soil microbes and then preferential decomposition of microbial derived C^52^, consequently causing break-down of macroaggregates into microaggregates and decomposition of OC therein^22^. The OC within microaggregates and silt-clay units was supposed to have lower turnover rates due to stabilization by organo-mineral interactions in bareland and cropland^18^, which played a significant role on C stabilization^13^. Xie *et al*.^53^ also found a similar change of aggregate associated C in loess soil when cropland was converted to fallow. Furthermore, the OC proportions of HF, FA, and HA fractions within microaggregate and silt-clay fractions were higher in cropland and bareland soils than those in grassland soil. This demonstrated greater C protection of aggregates in the fine fraction when substrates were limited as in bareland or cropland soil (Figs. 3,4). The reduced OC proportions within macroaggregate but increased OC proportions within microaggregate could be attributed to the lack of biological binding agents in cropland and bareland soils^6^.

## Conclusion

The 31-year contrasting land use patterns caused C redistribution within soil matrixes. The 0.25-2 mm macroaggregates and 0.25-0.053 mm microaggregates were considered to be the “preferential fraction” largely contributing to the accumulation of labile and recalcitrant OC in grassland and bareland soils, respectively. Converting cropland to grassland caused relatively higher proportions of OC sequestered within macroaggregates, which highlights the importance of reducing soil disturbance and enhancing plant residue inputs and potential microbial contribution for SOC accumulation, particularly in heavy and humin fractions within macroaggregates. However, the absence of plant-derived C inputs in bareland limited new OC resources and subsequently caused the depletion of macroaggregates and their associated OC. Our results demonstrated that the soil aggregate matrix served as a “sieve” causing C redistribution thereof, with land use changes influencing the C redistribution processes further.

## Materials and methods

### Study site

The experiment was established at the National Observation Station of Hailun Agro-ecology System, Heilongjiang province, China (47° 27′N, 126° 55′E) (Hailun Station). The experimental site is located in the center region of the Mollisols in Northeast China. The study site was very flat, with the slope less than 2 degree. The region has a temperate continental monsoon climate characterizing by simultaneously hot and rainy period from June to September in the growing season. Then the temperature sharply declined below 0 °C since October. The mean annual precipitation is 550 mm and mean annual air temperature is 1.5 °C. The soil is classified as Phaeozems^54^, which was developed from Loess parent material. The soil texture is predominantly silt and clay with the total content higher than 70%^14^ (Table 1). It is the unique climate condition, 2:1 clay nimerals (mainly vermiculite, smectite and illite), and the accumulation of relatively large amounts of organic matter in the soil, that forms the extremely fertile Mollisols with relatively high organic carbon content^28,29,55^.

### Experimental design

A long-term vegetation restoration experiment was established in 1985. The selected soil for this experiment was under crop cultivation for more than 100 years before simulated second restoration treatments began in 1985. The initial arable land was divided into three adjacent sections, simulated restored grassland, an overgrazed bareland, and a cropland under continuous soil tillage. The grassland (1120 m^2^ in size) was naturally restored without any fertilizer or tillage. *Leymus chinensis* gradually became the dominant grass species. The estimated shoot and root biomass were about 4–9 t ha^−1^ and 12–43 t ha^−1^, respectively (data not published). The bareland (1120 m^2^ in size) plot was maintained by periodically removing plants by hand hoeing to simulate the effects of overgrazing or extreme land degradation. The cropland (1050 m^2^ in size) was maintained as a continuously tilled arable soil under a 3-year soybean (*Glycine max* (L.) Merrill.) - maize (*Zea mays* L.) - wheat (*Triticum aestivum* L.) rotation since 1985 and was subject to a randomized block design using three fertilization management practices with four replicates (87 m^2^ of each treatment plot). In this study, only the cropped treatment without chemical fertilizer and organic amendments was selected to compare with bareland and grassland, under the consideration of eliminating the effects of chemical fertilization on soil aggregate. In cropland, all crop residues were removed from the plot following harvest to simulate the common practice locally. The soil underwent conventional tilling three or four times per year, including spring disking before planting, harrowing during growing season, and autumn moldboard plowing to a depth of 0.2 m after crop harvest.

### Soil sampling and analysis

The bareland and grassland field blocks were divided equally into four randomly distributed plots representing four field treatment replicates. Eight randomized soil cores (2.64 cm in diameter) per replicate were taken from a depth of 0–10 cm to make a composite soil sample after harvest of soybean on October 3, 2016. Sampling was performed in such a way so as not to break soil aggregates and to minimize compression. The soils were put into cloth bags, transported to the laboratory, part of the fresh soil samples were stored at 4 °C for soil microbial biomass carbon (SMBC), and another part were air-dried. A total of 12 field soil samples were collected. First, the air-dried bulk soil samples were broken down and then passed through an 8-mm sieve after manual elimination of visible plant litter and root residues prior to fractionation and analysis. Considering the small mass of archive soil representing the initial soil characters of the experiment in 1985, the 1985 soil sample was only used for water-stable aggregate mass fractionation, without further SOC fractionations within aggregates. The selected soil properties of the field soils and initial soil in 1985 were represented in Table 1.

Soil bulk density (SBD) was measured after drying soil cores at 105 °C for 48 h. SMBC was determined using the fumigation - extraction method^56^. The OC from the fumigated (24 h) and non-fumigated (control) soil were measured using a C analyzer (Shimadzu Model TOC-V). The SMB-C was calculated using a *k*_*EC*_ factor of 0.45^57^.

### SOC fractionation

The air-dried soil samples were used to physically fractionate the soil aggregates by wet sieving^14,23^. First, 100-g aliquots of soil samples that had been dried at 40 °C overnight were placed on the top of a column of three sieves measuring 2, 0.25, and 0.053 mm. Then, the sieves were immersed in deionized water for 3 min and gently moved up and down by hand for a total of 50 cycles over 2 min. The remains on the sieves and those passing through the 0.053 mm sieve were collected separately. The sieving process was repeated to obtain sufficient material for density and humic substance fractionation analysis for each aggregate size fraction. The final sieving was performed to determine aggregate size distribution, total organic C, and total N in each fraction after drying in an oven at 40 °C overnight, then weighed to determine the mass distribution among the aggregate size classes, namely, large macroaggregate (>2 mm), small macroaggregate (0.25–2 mm), microaggregate (0.053–0.25 mm), and soil silt-clay unit (<0.053 mm).

Density and humic fractionation of OC within different aggregate sizes were carried out according to the method described by Golchen *et al*.^58^, Yamashita *et al*.^3^, and Dou *et al*.^59^. First, a 10-g soil aggregate sample was put into a 100-mL centrifuge tube with 50 mL of sodium iodide (NaI) solution (*d* = 1.8 g cm^−3^) and left at 20 °C overnight. The following day, after centrifugation for 15 min at 3500 revolutions·min^−1^, the supernatant was passed through a membrane filter (0.45 μm) into a millipore vacuum unit. The fraction recovered on the filter was washed with a 50 mL 0.01 M calcium chloride (CaCl_2_) solution and 100 mL distilled water and then moved to a pre-weighed 50-mL beaker. The obtained fraction was dried at 50 °C to a constant weight and used as the free light fraction (fLF). Then, the residual soil was mixed with 50 mL of NaI solution, placed in an ice bath, and sonicated at 300 J·mL^−1^ using a probe-type ultrasonic disintegrator. After separation, the procedure was repeated. The obtained fraction was dried at 50 °C to a constant weight and used as the occluded light fraction (oLF). The leftover soil in the centrifuge tube was washed with distilled water until the water became clear, then oven dried at 50 °C to a constant weight and used as the heavy fraction (HF).

To attain humic substances within aggregates, a 2-mm-sieved aggregate sample was extracted using a solution of 0.1 M sodium pyrophosphate (Na_4_P_2_O_7_) and 0.1 M sodium hydroxide (NaOH) mixture (pH 13). The soil to extractant ratio was 1:6. The mixture was shaken in a thermostatic water bath oscillator for 1 h. The supernatant and particles were separated through centrifugation for 15 min at 3500 revolutions·min^−1^ to extract HA and FA. The extraction supernatant was filtered into a volumetric flask and diluted to 50-mL volume using distilled water. The supernatant was decanted, then acidified with 1 M sulfuric acid (H_2_SO_4_) to pH 1 and left for 24 h at room temperature to precipitate the HA. The HA fraction was dissolved by 0.05 M H_2_SO_4_ and 0.05 M NaOH. Titration was used to analyze HA and FA fractions. The residual soil particles (Humin) were analyzed with the VarioEL CHN elemental analyzer.

Total organic C contents in bulk soil, soil aggregates, and all OC fractions within different sized aggregates were analyzed using a VarioEL CHN elemental analyzer (Heraeus Elementar VarioEL, Hanau, Germany). The Mollisol is free of carbonates.

### Statistical analysis

Mean weight diameter (MWD) was calculated as an index of aggregate stability using the Eq. (1):

1 $$MWD=\mathop{\sum }\limits_{i=0}^{n}{P}_{i}{S}_{i}$$

where, *S*_*i*_ is the average diameter (mm) between (*i*–1)th and *i*th aggregate size and *P*_*i*_ is mass fraction of *i*th aggregate size.

The organic carbon content of different carbon fraction within each aggregate was calculated using the Eq. (2):

2 $${C}_{i}={C}_{ci}\times {P}_{i}\times {10}^{-2}$$

where *C*_*i*_ is the organic C content of *i*th carbon fraction (g kg^−1^), *C*_*ci*_ is the organic C concentration of *i*th C fraction (g kg^−1^), *P*_*i*_ is the proportion of *i*th C fraction within each aggregate.

The ratio of OC content in each C fraction within aggregate to total SOC was calculated as the proportion of OC in total SOC (Fig. 3).

One-way analysis of variance with Tukey honestly significant difference (HSD) as a post hoc was used for means separation of SOC and total nitrogen (TN) contents, aggregate fractions, density, and humic fractions among the three field treatments and the initial soil in 1985 at *P* < 0.05. Principal component analysis (PCA) of the combined data of organic C in aggregate and all sub-fractions within aggregate sizes was performed to visualize the development of soil as an aggregated function of these properties. The statistical analyses were performed using SPSS 16.0 for Windows.
